# Evaluability and feasibility of an intervention combining gaming and social activities to improve mental wellbeing and health behaviour in adolescents facing social challenges

**DOI:** 10.1186/s12887-026-07172-z

**Published:** 2026-06-20

**Authors:** Julie Christina Grew, Nina Gøtzsche, Lotte Broberg, Michaela Louise Schiøtz

**Affiliations:** 1 Center for Clinical Research and Prevention, Copenhagen University Hospital – Frederiksberg, Nordre Fasanvej 57, Hovedvejen 5, Frederiksberg, 2000 Denmark; 2 Department of Gynecology and Obstetrics, Slagelse Hospital, Faelledvej 14, Slagelse, 4200 Denmark; 3 Department of Clinical Medicine, University of Copenhagen, Blegdamsvej 3B, Copenhagen N, 2200 Denmark; 4 Department of Public Health, University of Southern Denmark, Campusvej 55, Odense M, 5230 Denmark

**Keywords:** Evaluability assessment, Feasibility, Acceptability, Evaluation design, Mental wellbeing, Loneliness, Adolescents, Gaming, Social activity, Physical activity

## Abstract

**Background:**

Adolescents in Denmark increasingly report poor mental wellbeing and health behaviour. Additionally, they spend less time being physically active and being with friends and more time on gaming and social media. The eHOOD intervention was developed to address physical and mental wellbeing of adolescents in vulnerable positions using their motivation for gaming to engage them in a community entailing physical and social activity, and education with peers. This study aimed to assess the evaluability of the eHOOD intervention, to develop an evaluation design, and to test the feasibility and acceptability of the evaluation design and the intervention.

**Methods:**

Evaluability assessment was performed by a working group in a series of workshop-style meetings involving a literature review, creation of a theory of change, and preparation of an evaluation plan including outcome measures, instruments, and data collection procedures. Subsequently the evaluation design and the intervention were tested in a single arm feasibility study. Participants were 13 adolescents aged 13–16 years. The participants met with a coach and a local pedagogue in an after-school club four hours a week for 25 weeks practicing gaming, engaging in physical and social activities, and learning about healthy lifestyle habits through education and communal cooking and dining.

Feasibility and acceptability of outcome measures, instruments, and data collection procedures were assessed, and initial changes in outcome measures were measured. Recruitment and retainment of participants were monitored, and participants’ motivation for and benefits of participating were explored. Data were collected with qualitative and quantitative methods.

**Results:**

An evaluation plan was developed and found to be suitable for use in the subsequent feasibility study. The evaluation design was overall feasible and acceptable, although several important adjustments to measurements and data collection procedures were identified. Initial changes in wellbeing were observed and the World Health Organization-Five Well-Being Index (WHO-5) was deemed relevant as a primary outcome in future evaluation. The intervention was found feasible and acceptable among participants in the present format.

**Conclusions:**

This combined evaluability assessment and feasibility study suggests that the eHOOD intervention is ready for larger-scale evaluation, although refinements to outcome measures and data collection procedures are warranted before effectiveness can be evaluated.

**Trial registration:**

ClinicalTrials.gov, March 23, 2026. Identifier NCT07489365. Retrospectively registered.

**Supplementary Information:**

The online version contains supplementary material available at https://doi.org/10.1186/s12887-026-07172-z.

## Introduction

Adolescents in Denmark increasingly have poor mental wellbeing and unhealthy lifestyles. According to a recent study based on register data from school nurses’ examinations of school children, at least one concern regarding general wellbeing was raised for 14.2% of 14-year-olds and 12.3% of 15-year-olds [24]. In the Danish version of The Health Behaviour in School-aged Children (HBSC) from 2022, consisting of survey data from children aged 11, 13, and 15 years, 12% of girls stated they frequently or very frequently experience loneliness, and 30% of girls reported low mental wellbeing. Furthermore, comparing these findings with the past 10 to 20 years, significant negative trends are observed across a wide range of mental health parameters [19]. Comparable patterns are seen across Europe and North America [6, 25]. As for lifestyle habits, only one-third of girls and around half of boys met the Danish recommendations for physical activity and intake of fruit and vegetables [19]. In school nurses’ registers [24] almost 17% of the adolescents aged 14–15 years were classified as overweight following sex- and age-specific body mass index (BMI) cut-offs [5]. In addition to spending less time being physically active, adolescents also spend less time on face-to-face activities with friends and more time on social media and gaming. According to the Danish HBSC, time spent face to face with friends has significantly decreased in recent years, and 15-year-old boys use on average 159 min per day gaming and 141 min per day on social media. Correspondingly, 15-year-old girls use on average 70 min per day gaming and 190 min per day on social media [19]. Across all the countries in the international HBSC, one-third of pupils aged 11–15 years reported gaming online every day, and 22% reported gaming online for at least four hours per day [1].

Although the potential harm of adolescent use of digital technology is often highlighted in the public debate, research has shown both positive and negative consequences of the comprehensive use of screens among adolescents. For example, a systematic review found that the use of internet technology increased adolescents’ sense of connection to friends while at the same time increasing anxiety and loneliness [31]. A more recent scoping review found positive effects of adolescent use of gaming, social media, and the internet regarding social support, social connection, and communication skills [12]. Particularly for adolescents in vulnerable social positions, there is concern about the potential negative consequences of digital technology use. For instance, research has shown an association between the daily usage of digital technology and poor mental health [11], although no cause-and-effect relationship has been established. A review found that while the digital practices of vulnerable adolescents enable social support and connection, they may also expose them to a range of negative experiences [15].

The implications of adolescents having less physical contact with friends and peers are yet to be fully understood. What we do know is that health and wellbeing in childhood and adolescence have implications for educational achievement and mental wellbeing in adulthood and, thus, overall life achievements [14]. In a survey among Norwegian adolescents [7], the risk of high school dropout was associated with various health problems such as psychological distress, insomnia, concentration difficulties, and overweight. Untreated mental health issues in adolescence, such as anxiety and depression, are shown to often continue into adulthood [21].

In acknowledgement of adolescents’ extensive use of digital technology while at the same time recognizing a potential negative impact on their mental health, the eHOOD intervention was developed in 2018 aiming to increase mental and physical health and wellbeing among adolescents facing social challenges. eHOOD combines gaming, physical activity, health education, and social interaction in safe and familiar surroundings, fostering the development of skills and positive social relations with peers and adults–elements of organised leisure communities that have been shown to support mental wellbeing [16]. It uses adolescents’ motivation for improving their gaming skills to teach and engage them in healthy lifestyle, building on a possible link between physical fitness and cognitive skills associated with gaming skills [20, 30]. The eHOOD intervention has already been used in several Danish municipalities but has never been systematically evaluated.

On basis of this, the objective of this study was threefold: (1) to assess the evaluability of the eHOOD intervention, (2) to develop an evaluation design, and (3) to assess the feasibility of the evaluation design and the intervention for use in future, larger-scale evaluations.

Our research questions were:

1. What is an appropriate evaluation design for testing the eHOOD intervention?
2. To what extent is the evaluation design feasible in terms of outcomes, measurements, instruments, data collection procedures, and participant acceptability?
3. To what extent is the intervention feasible and acceptable in terms of recruitment, retention of participants, and participant satisfaction and engagement?

## Methods

The study consists of an evaluability assessment followed by a feasibility study in a before-after single arm design, implying that the study was conducted without a control group and with measurements before and after the intervention. In the following, the methods and results of the two studies will be reported separately.

In accordance with the guideline on reporting non-randomised pilot and feasibility studies [17], this feasibility study adheres to the CONSORT Extension to Randomised Pilot and Feasibility Studies [9] except that items concerning randomization were omitted.

### Evaluability assessment

The evaluability assessment was conducted prior to intervention start, between August and December 2022. It was carried out through a series of workshop-style meetings and site visits to the location of the planned feasibility study, an after-school club in a Danish municipality. The process of the evaluability assessment was inspired by Brunner, Craig, and Watson [4] and conducted in five steps:

1. In the first step, a working group to conduct the evaluability assessment was established. The first author served as the principal investigator, leading the working group. The participants included a manager from Sincera (a Danish private limited liability company), where the intervention was developed; the eHOOD coach; a data analyst from Impactly (a Danish private limited liability company) that delivered an online platform tested for data collection and analysis in the study; a local pedagogue from the after-school club; and a consultant from SENS Innovation (a Danish private limited liability company) that delivered the SENS motion^®^ accelerometers tested for collecting data on physical activity and sleep in the study.
2. In the second step, the first author, the Sincera manager, the eHOOD coach, and the Impactly data analyst constructed an agreed-upon theory of change based on previous experiences with the intervention and a literature review. The theory of change was continuously refined throughout the study and served as the basis for the development of the evaluation plan.
3. In the third step, the first author reviewed the literature regarding the use of outcomes, instruments, and data collection procedures in previous research on adolescents and identified possible data sources. This was done with support from the Sincera manager and the Impactly data analyst.
4. In the fourth step, the first author and the Impactly data analyst made recommendations for evaluation and a draft version of an evaluation plan.
5. In the fifth step, the working group discussed and revised the evaluation plan. The first author had the final say in decisions regarding the evaluation.

**Fig. 1:**
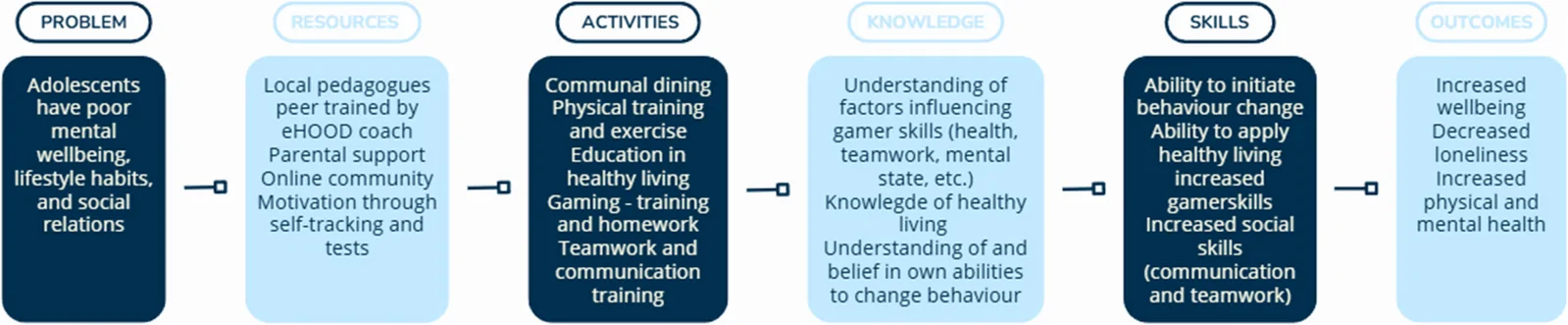
Theory of change

The outcomes of the evaluability assessment were a theory of change and an evaluation plan outlining outcomes, measurements, data sources, and data collection procedures for the subsequent feasibility study. The evaluation plan is described in the section *Evaluation plan* below and presented in Fig. 3. The theory of change is shown in Fig. 1.

The evaluability assessment resulted in an agreement to proceed to the feasibility study in which the evaluation plan was applied.

### Before-after single arm feasibility study

#### Setting

The intervention was offered in a municipal after-school club in the vicinity of Copenhagen, Denmark.

#### Participants

The target population of the eHOOD intervention was adolescents in 7th to 10th grade (corresponding to lower secondary education) with psycho-social challenges. Potential participants were identified by a local pedagogue, who collaborated closely with schoolteachers and after-school club pedagogues. Altogether, the group possessed profound knowledge of the adolescents in the area. Based on a professional assessment of individual needs and anticipated benefits, adolescents with psycho-social challenges such as anxiety, loneliness, school refusal, and being a victim of bullying, were invited to participate. Adolescents who were deemed unable to participate meaningfully in a social community were excluded. Recruitment began in October 2022 and continued until 12–15 potential participants were identified, a target set by the eHOOD team based on prior experience. This goal was successfully achieved in December 2022. Prior to intervention start, eligible participants and their parents were invited to an information meeting hosted by the eHOOD team. The meeting was held one evening at the after-school club where the intervention was going to be tested.

#### Intervention

**Fig. 2:**
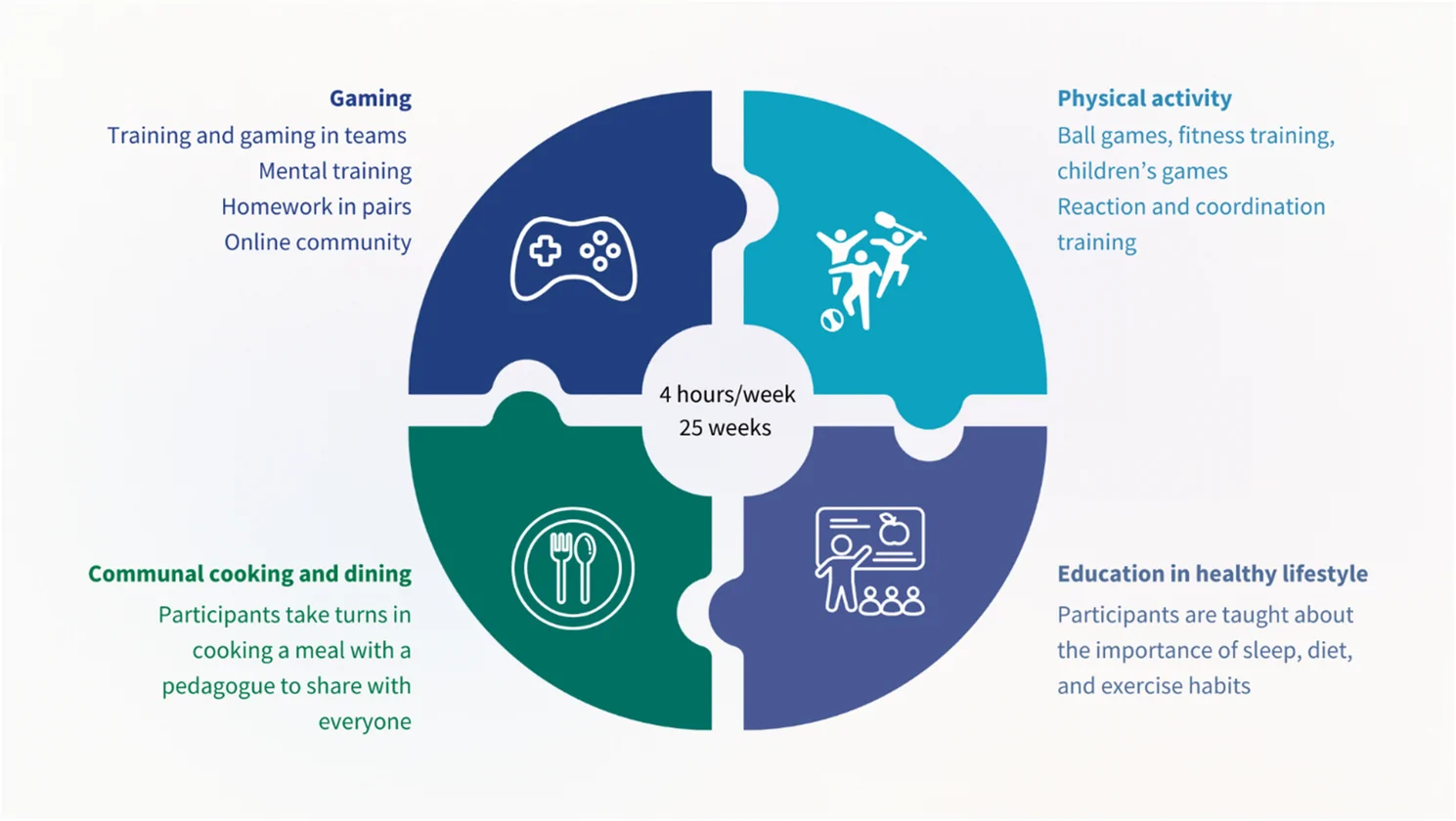
Overview of content of the eHOOD intervention

The eHOOD intervention consisted of weekly meetings for the adolescents for 25 weeks. Participants meet up with their eHOOD coach and one or more local pedagogues one evening from five to nine PM every week. Each meeting contained physical activity, preparation and intake of dinner, education, and gaming with instruction. Physical activities included ball games such as football, basketball, and table tennis, fitness training, and traditional children’s games depending on local facilities and participants’ preferences. Coordination and reaction training were included. Educational sessions included teaching healthy dietary habits, sleeping habits, and exercise habits, focusing on the importance of taking care of one’s body and staying fit to be able to perform well in gaming. Gaming sessions were structured and led by the eHOOD coach and contained both training and gaming in teams. Further, participants were paired in teams and were given small assignments to be performed together between the weekly meetings. Homework was meant to form and nurture new relationships and invite teammates to stay in contact outside the physical meetings. As parts of the social and educational activities, communal cooking and dining were included. Participants took turns preparing and serving dinner for their peers together with the local pedagogue. Figure 2 illustrates the elements of the eHOOD intervention.

#### Organisation

The eHOOD team managed communication with the municipality and oversaw the implementation of the intervention. Impactly facilitated survey data collection through its proprietary platform, and SENS Innovation handled the collection of accelerometer data. Importantly, the research team retained exclusive responsibility for evaluating the data and interpreting the findings. None of the collaborating partners had any involvement in the analysis, the results, or their presentation, ensuring the independence and integrity of the research process.

#### Evaluation plan

**Fig. 3:**
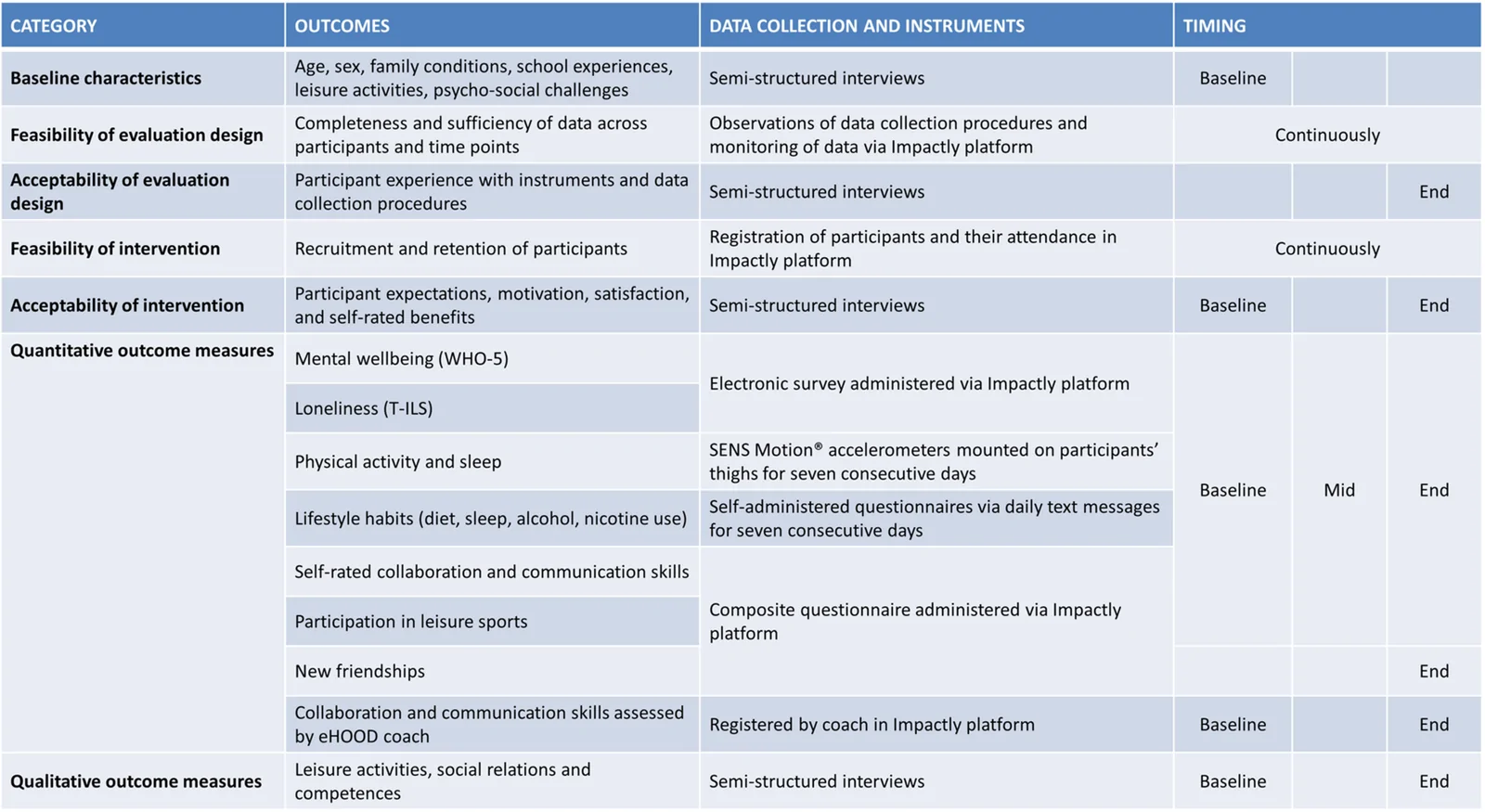
Outcomes, instruments, data collection procedures, and timing of measurements

Feasibility outcomes were prespecified during the evaluability assessment and concerned the feasibility and acceptability of the evaluation design and the feasibility and acceptability of the intervention. The selection of feasibility domains, outcomes, instruments, and progression criteria was informed by published guidance on feasibility and pilot studies in public health interventions [13, 22, 23, 28]. These sources informed both the structure of the evaluation plan and the selection of feasibility outcomes to be tested in the subsequent feasibility study. Quantitative and qualitative outcome measures were selected for initial testing of detectable changes to be used as outcomes in a future evaluation of the intervention. Priority was given to brief and acceptable measures considered suitable for use in adolescent populations [18, 19]. Validated instruments were selected where possible to support future scalability and comparability across studies and included the 5-item World Health Organization Well-Being Index (WHO-5) and the Three-Item Loneliness Scale (T-ILS). The WHO-5 includes five items each scored from 0 to 5. Thus, raw scores range from 0 to 25 and are multiplied by 4 to transform it to a percentage scale from 0 to 100, where the max score corresponding to optimal wellbeing is 100, 50–70 points corresponds to the normal range of wellbeing, and a score under 50 points corresponds to a wellbeing below the normal range, equalling risk of depression [29]. The T-ILS includes three items each scored from 1 to 3. Total scores range from 3 to 9, where 9 corresponds to the highest level of loneliness. There is no consensus on a threshold value, but in a Danish study of loneliness among adolescents, they used a score of 5–6, equalling moderate loneliness, and a score of 7–9, equalling severe loneliness [18]. Two questionnaires developed for this study (see Appendix A) covered lifestyle habits and self-rated communication and collaboration skills using items from the Danish HBSC [19] and the annual wellbeing survey in Danish public schools [8]. The latter questionnaire was supplemented with a small number of self-constructed items concerning participation in organised sports activities and new friendships; subjects that we could not find covered in existing questionnaires. Figure 3 visualises the selected feasibility outcomes as well as the quantitative and qualitative outcomes, instruments, and data collection procedures that were tested, and the timing of measurements.

Progression to a future definitive trial was guided by predefined feasibility criteria, including the ability to recruit the intended number of participants within the planned timeframe, acceptable retention with no dropouts, and the feasibility and acceptability of outcome measures and data collection procedures. These criteria were not used as strict thresholds but to inform necessary adjustments and the overall judgement of readiness for a larger-scale evaluation.

#### Data analysis

Quantitative data were analysed descriptively to identify initial changes in selected outcomes. For survey data collected using the Impactly platform, changes in mean values were calculated and reported. Accelerometer data were categorized into the following categories: inactivity (lying/sitting), moderate-intensity activity, high-intensity activity, sleep (calm and restless), and number of steps. For each category, daily minutes were calculated, and weekly averages were derived for each individual participant. At the group level, changes in mean values were calculated to explore trends. Qualitative data collected via interviews and observations were analysed thematically with inspiration from Braun and Clarke [2, 3].

## Results

### Participants and data collection

All participants in the eHOOD intervention were part of the feasibility study. The participants were 13 adolescents aged 13–16 years with a median age of 14 years at intervention start. The intervention ran from January 2023 to August 2023. Baseline measurements were conducted in February 2023, mid-point measurements in June, and end measurements in August 2023. The feasibility study was completed as planned with no early termination. Participants’ self-reported baseline characteristics are shown in Table 1.

**Table 1:** Participants’ self-reported baseline characteristics

Participant characteristic	*N* (%)	Range	Median
Age		13–16	14
Gender			
- Female	4 (31)		
Family conditions			
- Living with only one parent	5 (39)		
School experiences			
- Changed school or class at least once due to social issues	7 (54)		
Leisure activities			
- Participates in organised sports activities at least once a week	7 (54)		
- Online gaming is the main leisure activity	8 (62)		
Psycho-social challenges			
- Lack friends and peers to hang out with	12 (92)		
- Diagnosed with anxiety, dyslexia, ADHDᵃ, and/or ASDᵇ	7 (54)		

ᵃAttention Deficit Hyperactivity Disorder

ᵇAutism Spectrum Disorder

Table 2 shows which outcomes were measured, how and when, and the number of participants for whom complete data were collected at each point of measurement.

**Table 2:** Outcomes, instruments, data collection procedures, and number of participants with complete data

Outcome	Instrument	Data collection procedure	Baseline (*N*)	Mid (*N*)	End (*N*)
Mental wellbeing	WHO-5	Electronic survey administered by researchers	13	12	8
Loneliness	T-ILS	Electronic survey administered by researchers	13	12	8
Physical activity	Accelerometer	Coach mounts on participants	9ᵃ	8ᵇ	2ᶜ
Sleep					
Food intake	Composite questionnaire	Self-administered electronic survey via text message	7	3ᵈ	2ᵉ
Sleep habits					
Alcohol and nicotine use					
Self-rated collaboration skills	Composite questionnaire	Electronic survey administered by researchers	13	12	8
Self-rated communication skills					
New friendshipsᶠ					
Participation in leisure sports					
Self-rated benefits of eHOOD		Qualitative interview by researchers			8
Collaboration and communication skills	Composite questionnaire	Electronic survey administered by coach	12		13

ᵃ11 participants accepted to wear an accelerometer, but two took it off or lost it before complete data were collected

ᵇOnly four of those participants who wore an accelerometer at mid-measurements also wore it at baseline

ᶜFour participants accepted to wear an accelerometer, but two took it off or lost it before complete data were collected

ᵈFive participants responded to the survey, but only three filled it out each day of the week

ᵉEight participants responded to the survey, but only two filled it out each day of the week

ᶠOutcome only collected at end measurements

### Feasibility and acceptability of evaluation design

#### Measurements, instruments, and data collection procedures

Administration of electronic surveys for measuring WHO-5, T-ILS, and self-rated communication and collaboration skills was planned to be a task for the coach during eHOOD sessions. This task turned out to disturb the flow of the sessions for the coach, resulting in missing data collection. To solve this problem, the first and second authors went to the after-school club and filled in the surveys with the participants instead. The delay means that baseline measurements were done in April and not in February as expected. All participants willingly and easily answered the surveys administered by the researchers, and useful data were collected.

Measuring physical activity and sleep with accelerometers was challenging in the target group. Out of 13 participants, 11 accepted to wear an accelerometer at baseline, and two did not want to wear it. Of the 11 who wore it, two lost or removed it before seven days had passed. Thus, complete data were collected for nine participants. At mid-measurements eight participants wore an accelerometer for seven consecutive days, but three of them did not wear an accelerometer at baseline. At end of intervention, only four participants accepted to wear an accelerometer again, and two lost it or removed it. Therefore, complete data were only collected for two participants and no data on physical activity and sleep are therefore reported here.

The self-administered survey sent to the participants by text message daily for seven consecutive days was acceptable to participants, and questions were easy to answer. Nevertheless, no useful data on lifestyle habits were collected, because only three participants answered each of the seven consecutive days at mid-measurements and two participants at end-measurements. Interviews revealed that most participants did not understand the purpose of answering homonymous questions every day for several days. Further, items regarding alcohol and nicotine use did not generate useful data due to unsuitable wording.

Collaboration and communication skills were relevant and acceptable measures to both participants and coach, and they generated useful data. However, participants’ self-assessments and the coach’s assessments were not directly comparable due to differences in item wording and response categories. While both suggested improvements over time, the measures appeared to capture somewhat different aspects of communication and collaboration skills.

#### Initial changes in outcomes

The intervention showed potential regarding its ability to increase participants’ wellbeing, reduce loneliness, support participants in building new relationships, and strengthen participants’ social skills. To measure the changes in WHO-5 and T-ILS we used baseline measurements and mid-measurements due to low response rates at end measurements. The average WHO-5 score of the eHOOD participants increased from 46.8 at baseline to 57.5 at mid-measurements, indicating a clinically relevant improvement of wellbeing of 10 + points [29]. The average T-ILS score declined from 5.2 to 4.2, indicating reduced loneliness.

All participants reported that they made new friends, and seven out of the 13 participants reported that they had several or many new friends by the end of the intervention.

**Fig. 4:**
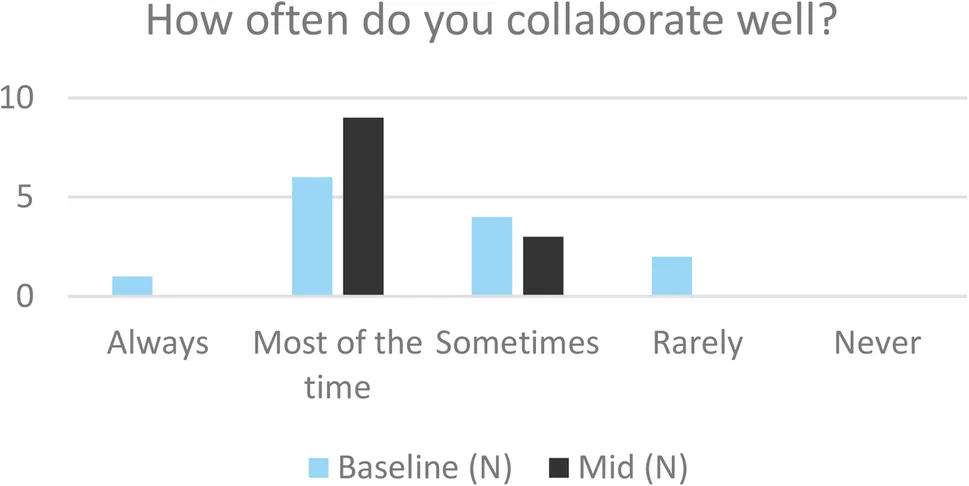
Participants’ self-rated collaboration skills

To measure changes in self-rated collaboration skills we used baseline and mid-measurements due to a low response rate at the final measurements. As seen in Fig. 4, at baseline six participants said they collaborate well most of the time, and two said they rarely collaborate well. By mid-measurements, nine participants said they collaborate well most of the time, and the remaining three said they sometimes collaborate well.

**Fig. 5:**
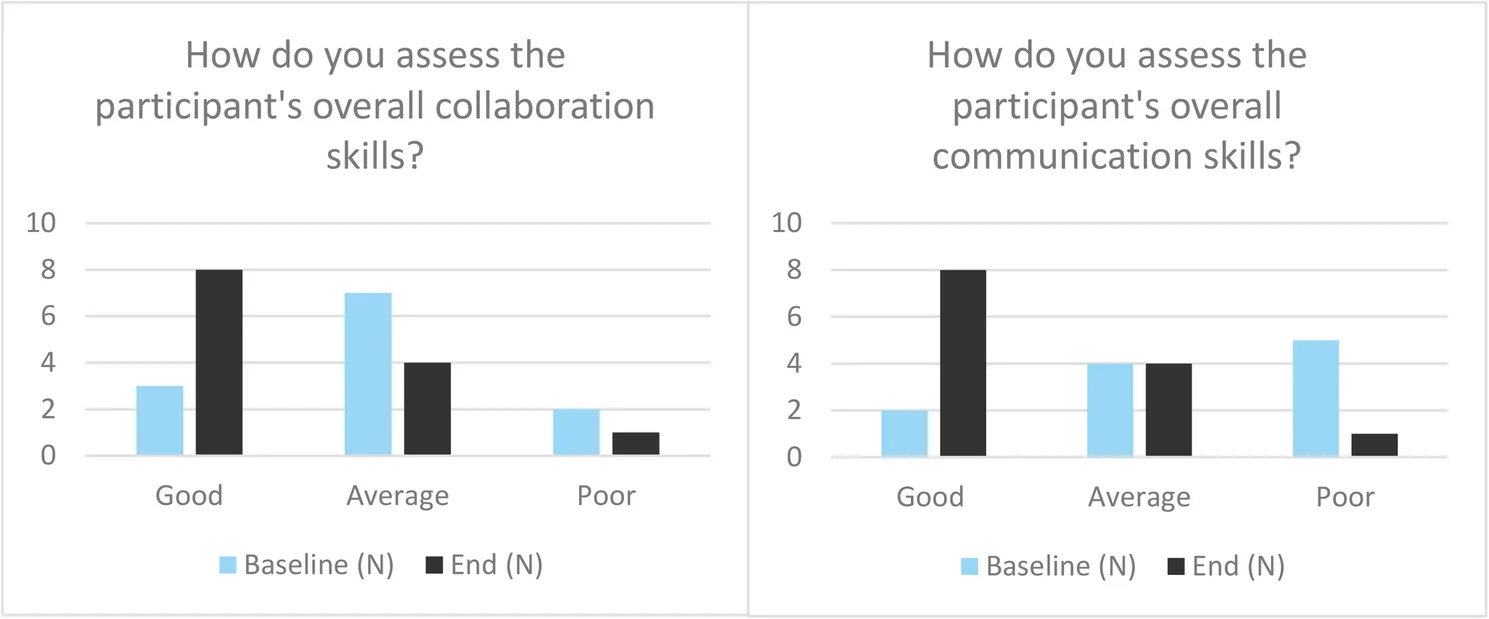
Assessment of participants’ collaboration and communication skills

Other items were used for eHOOD coach’s assessment of participants’ collaboration and communication skills. Figure 5 shows that, according to the coach, three participants had good collaboration skills at baseline and eight participants at the end of the intervention. Similarly, two participants had good communication skills at baseline according to the coach and by the end of the intervention, the number had increased to eight participants.

### Feasibility and acceptability of intervention

We investigated participants’ expectations and motivation for participating in eHOOD and their self-rated benefits of participating. Most participants said that gaming was attractive to them and that they perceived eHOOD as an opportunity to practice meeting new friends. Many participants expressed hope of making new friends by joining eHOOD, especially friends to meet with physically rather than virtually, as many of them primarily met friends online. Some participants said it took courage to sign up for eHOOD because they did not know the other participants in advance. Some told us about school fatigue and expressed hope that eHOOD could strengthen their self-discipline in relation to school and homework. Some participants expressed problems with raging and foul language during gaming and wished to gain more control over their language and behaviour. Others wanted more control over their gaming consumption and to practice prioritizing their time. Overall, motivational factors were the desire to increase social skills, self-discipline, and self-control. Many participants mentioned that cooking, communal dining, and physical activity made eHOOD fun and special. Almost all participants highlighted the combination of activities as something extraordinary because it built a strong social community, and the community retained their interest in eHOOD. Hence, gaming and other social activities attracted the participants to begin with, but the community made them stay. The present study did not systematically assess whether homework assignments achieved their intended purpose of strengthening relationships between participants outside weekly meetings. As for recruitment and retention, a sufficient number of participants was recruited within the planned period, and there were no dropouts during the intervention period. No harms or unintended effects related to participation in the intervention were observed or reported during the study period.

## Discussion

### Interpretation

Relevant adjustments of the evaluation design were identified to optimise data collection and measurement relevance for use in future evaluation and similar interventions targeting adolescents, specifically:

#### Outcome measures

The WHO-5 proved feasible and acceptable and showed positive trends in change from baseline to mid-measurements, as the average score increased slightly more than 10 points indicating a clinically relevant change. The average score was well below the mean score of 70 in the general population in Denmark [29] but within the normal range of 40–80 of adolescents aged 13–15 years in the HBSC [19]. Our findings regarding participants’ sense of connectedness fostered by their participation suggest that eHOOD, as an organised leisure activity, has the potential to support the mental wellbeing of participants [16]. On this basis, we believe that mental wellbeing measured with the WHO-5 is a relevant primary outcome in future evaluation.

#### Data collection procedures

Our difficulties collecting complete data from all participants is an important learning for future evaluations of interventions targeting adolescents in vulnerable positions. The use of accelerometers proved less suitable in the particular group of participants. Other Danish studies have used SENS Motion accelerometers for 11-year-old children but have failed to report participation and retention rates [26] or have yet to report the results [27]. Thus, it is not clear whether our lack of success was due to the ages and the psycho-social challenges of the target group—all of which possibly blurred their interest in contributing to our data collection—or whether we could have attained sufficient data if our sample size had been bigger. Alternative approaches, such as integrating physical activity questions into surveys, could enhance data completeness and reliability. Additionally, clear communication about the importance of daily responses to surveys can address gaps in participant adherence. In a recent study of school children parental involvement in the administration of text message surveys was used to secure appropriate response rates (Stage et al., unpublished research), which highlights the need for such adaptations. Despite the practical challenges related to accelerometer-based measurements, physical activity and sleep remain relevant outcomes in future evaluations of the intervention given the recognized interrelationship between physical activity, sleep, sedentary behaviour, and mental wellbeing in children and adolescents (see, for example, [10]).

#### Supplemental measures

Including a measure for self-efficacy could provide a broader understanding of the intervention’s impact, as the development of self-efficacy has proved to be vital to support mental wellbeing in organised leisure activities for adolescents [16].

#### Ethical considerations

As defined by the “*Danish Act on Research Ethics Review of Health Research Projects*” Sect. 2, the project does not constitute a health research project, because the intervention was implemented independently of the research project and participation in the intervention was not contingent on study participation. Therefore, formal ethical approval was not required. Nevertheless, ethical considerations related to informed consent, participation of minors, and handling of wellbeing data remained central throughout the study. Future effect evaluations of the intervention may require formal ethical approval depending on the study design and level of researcher involvement.

### Limitations

The small sample size and missing data from mid- and end measurements pose limitations that necessitate caution in interpreting the results. The missing data were largely attributed to unstable attendance around the summer holidays, rather than participant disengagement, as evidenced by the absence of dropouts. Another limitation is the delay in some of the baseline measurements, which were done in April instead of February. This means that changes in mean values were observed within a short time interval, potentially reducing the ability to distinguish between natural variations and changes associated with the intervention. Seasonal variation may also have influenced some outcomes, as baseline and follow-up measurements were conducted during different times of the year with varying daylight hours and weather conditions potentially affecting physical activity, mood, and social behaviour.

Further, the intervention was only tested in a single site. Thus, our findings stem from one particular after-school club with specific pedagogues employed, distinct physical surroundings, and other unique characteristics. Involving more sites would have provided broader insights by capturing the diverse cultures, resources, and operational practices of different after-school clubs.

The identified challenges highlight the need for refined strategies in future evaluations to mitigate data gaps.

## Conclusions

### Summary of findings

The evaluability assessment indicated that the eHOOD intervention was sufficiently developed to proceed to feasibility testing using the proposed evaluation plan. The feasibility study further suggested that key elements of the evaluation design were feasible and acceptable, although several refinements to outcome measures and data collection procedures are needed before effectiveness evaluation. The feasibility study yielded valuable insights into the appropriateness of outcomes, instruments, and data collection procedures. An appropriate evaluation design for the eHOOD intervention appears to be a design combining validated self-reported measures of mental wellbeing and loneliness with qualitative exploration of participant experiences and engagement. Researcher-administered electronic surveys proved feasible and acceptable, whereas accelerometer-based assessments and repeated daily surveys were less suitable in this target group.

The intervention itself proved feasible and acceptable regarding recruitment and retainment of participants. High satisfaction expressed by participants highlighted the acceptability of the intervention, particularly regarding the combination of activities and the sense of social community fostered among participants. No dropouts occurred during the intervention period, reflecting strong engagement and commitment. To our knowledge, no similar interventions that have been evaluated exist. Improvements in wellbeing and loneliness should be interpreted with caution, as they were based on a small feasibility sample and limited endline data.

### Directions for future evaluation

Future studies should aim to:

- Use aligned wording and response categories in participant- and coach-reported measures of communication and collaboration skills to facilitate meaningful comparison.
- Enhance data collection procedures by replacing accelerometers with text-message-based surveys and ensuring participants understand their importance.
- Introduce supplemental measures, such as self-efficacy and school absenteeism, to capture additional dimensions of intervention impact.
- Conduct follow-up assessments post-intervention to evaluate long-term effects on wellbeing and social dynamics.

While our findings are based on a single feasibility study with a small sample, the study offers methods and insights regarding evaluability assessments and feasibility testing of interventions within similar target groups for researchers undertaking related work. By addressing the limitations and implementing the recommended adjustments, future studies can build on this foundation to yield more comprehensive and generalisable findings.

## Supplementary Information


Supplementary Material 1.


## Data Availability

The trial protocol and the datasets used and analysed during the current study are available from the corresponding author on reasonable request.

## Abbreviations

ADHD: Attention Deficit Hyperactivity Disorder
ASD: Autism Spectrum Disorder
HBSC: Health Behaviour in School-Aged Children
T-ILS: Three-Item Loneliness Scale
WHO-5: World Health Organization-Five Well-Being Index
