# Supplementary material for: Evaluability and feasibility of an intervention combining gaming and social activities to improve mental wellbeing and health behaviour in adolescents facing social challenges
Source: BMC Pediatr. 2026 Jun 20;26:821. doi: 10.1186/s12887-026-07172-z (PMC13536880; doi:10.1186/s12887-026-07172-z)
Supplement: Supplementary file 1 [file 12887_2026_7172_MOESM1_ESM.docx]

**Questionnaire on lifestyle habits**

|  | Yes | No |
| --- | --- | --- |
| Have you eaten breakfast today? |  |  |
| Have you eaten one or more meals with your family today? |  |  |
| Have you smoked tobacco and/or used snuff/chewing tobacco/vaporizer/nicotine pouches today? |  |  |
| Have you drunk alcohol (e.g. beer, wine, spirits, shots, alcopops) today? |  |  |

|  | Yes, several times | Yes, one time | No |
| --- | --- | --- | --- |
| Have you eaten fruits and/or vegetables today? |  |  |  |
| Have you eaten food from a burger bar, pizzeria and/or shawarma bar today? |  |  |  |

|  | Very tired | A little tired | Not tired |
| --- | --- | --- | --- |
| How tired did you feel this morning? |  |  |  |

|  | Yes | No | I did not have homework |
| --- | --- | --- | --- |
| Have you done your homework today? |  |  |  |

**Questionnaire on self-rated communication and collaboration skills, new friendships, and participation in leisure sports**

|  | Always | Most of the time | Sometimes | Rarely | Never |
| --- | --- | --- | --- | --- | --- |
| How often do you collaborate well? |  |  |  |  |  |
| How often do you communicate well? |  |  |  |  |  |
| How often do you speak rudely to your fellow players/teammates? |  |  |  |  |  |

|  | Yes, many | Yes, some | Yes, a few | No |
| --- | --- | --- | --- | --- |
| Have you made any new friends in eHOOD? |  |  |  |  |

|  | Yes | No |
| --- | --- | --- |
| Do you do recreational sports at least once a week? |  |  |
